# Associations between intimate partner violence and adverse birth outcomes during pregnancy: a systematic review and meta-analysis

**DOI:** 10.3389/fmed.2023.1140787

**Published:** 2023-05-17

**Authors:** Cancan Guo, Mengtong Wan, Yue Wang, Peijie Wang, Marissa Tousey-Pfarrer, Haoyang Liu, Liangming Yu, Lingqi Jian, Mengting Zhang, Ziqi Yang, Fenfen Ge, Jun Zhang

**Affiliations:** ^1^West China School of Medicine, Sichuan University, Chengdu, China; ^2^School of Medicine, Shanghai Jiao Tong University, Shanghai, China; ^3^Mental Health Center, West China Hospital, Sichuan University, Chengdu, China; ^4^Center of Public Health Sciences, Faculty of Medicine, University of Iceland, Reykjavík, Iceland; ^5^School of Education, Tianjin University, Tianjin, China

**Keywords:** intimate partner violence, preterm birth, low birth weight, stillbirth, meta-analysis

## Abstract

**Background:**

Intimate partner violence (IPV) has been associated with an elevated risk of multiple adverse birth outcomes, yet little is known about how specific IPV influences adverse birth outcomes. The aim of this study was to examine the association between IPV during pregnancy and adverse birth outcomes (i.e., preterm birth, low birth weight, and stillbirth).

**Methods:**

Systematic searches were conducted using four databases: EMBASE, Web of Science, PubMed, and CINAHL for observational studies published from 1 January 2011 to 31 August 2021. Two reviewers independently carried out the literature search, study selection, data extraction, assessment of the study, and risk of bias assessment; disagreements were resolved by a third reviewer. A random-effect model was used to calculate the odds ratio (OR) with a 95% confidence interval (CI) for preterm birth, low birth weight, and stillbirth. *I*^2^ statistic accompanied by chi-square *p*-value was used to assess heterogeneity, and funnel plot and Peter's test were used to assess publication bias.

**Results:**

In total, 23 studies met the inclusion criterion. IPV was associated with preterm birth (OR = 1.84; 95% CI: 1.37–2.49; *I*^2^ = 88%), low birth weight (OR = 2.73; 95% CI: 1.66–4.48; *I*^2^ = 95%), and stillbirth (OR = 1.74; 95% CI: 0.86–3.54; *I*^2^ = 64%). We attained comparable results among all specific IPV including physical, sexual, emotional, and mixed.

**Conclusion:**

Intimate partner violence and specific IPV during pregnancy were significantly associated with adverse birth outcomes, especially for physical IPV. An urgent need for greater action to prevent or intervene in IPV during pregnancy is warranted.

**Systematic review registration:**

CRD42021282936, https://www.crd.york.ac.uk/prospero/.

## 1. Introduction

Intimate partner violence (IPV) is a major and common public health problem in society globally (1). IPV refers to physical, sexual violence, and psychological aggression by a current or former intimate partner (2). A national prevalence estimate showed that approximately 33% of Americans have experienced IPV at some point in their lifetime (3). Specifically, an international survey conducted in 2005 by the World Health Organization (WHO) reported that the prevalence of IPV during pregnancy ranged from 4 to 12% (1). Whether IPV occurs during pregnancy or not, it could increase the risk of physical and mental disorders in both women and their children.

Previous studies have explored the associations between IPV and adverse birth outcomes including preterm birth, low birth weight, and stillbirth (4, 5). The relationship between IPV and adverse birth outcomes is complex. Although most researchers have found a positive association between IPV and adverse birth outcomes, some have not (6). For example, both Hill et al. (7) and Berhanie et al. (8) found an association between IPV and preterm birth, while Laelago et al. (6) did not. Pallitto et al. found no significant association between abuse and low birth weight after adjusting for confounding factors (9). Methodological limitations including search strategy, respondent selection, and mode effects may prevent drawing conclusions.

Various types of IPV have been associated with adverse birth outcomes, including physical and sexual IPV (10). Previous systematic reviews have been focused on this topic, with some defining violence as physical or sexual IPV, and found that IPV was strongly related to low birth weight as well as preterm birth, while intrauterine growth restriction was not significantly associated with IPV (7). There has been relatively better research attention on emotional or physiological IPV because of their less visible immediate impact. However, evidence is also emerging for the adverse birth outcomes of emotional or psychological-based IPV (11, 12).

We conducted a comprehensive and rigorous meta-analysis to explore the association between IPV and adverse birth outcomes. Our main aims were to: (1) evaluate the association between IPV during pregnancy and three adverse birth outcomes: preterm birth, low birth weight, and stillbirth, which were leading causes of neonatal morbidity and mortality; (2) explore the relation of specific types of IPV with adverse birth outcomes.

## 2. Inclusion criteria

The review included studies reported on IPV during pregnancy considering every pregnant woman as a population. The exposure to IPV was defined as experiencing any physical, sexual, or emotional abuse perpetrated by a current or former partner. We defined women who experienced more than one type of IPV at the same time as mixed IPV in the present study. Women who reported not experiencing IPV were considered as a control in this review. The outcome of the review included preterm birth, low birth weight, and stillbirth. Preterm birth refers to a baby born before 37 weeks of gestation, whereas a low birth weight is referring to a baby born with a weight of < 2,500 grams. A stillbirth is defined as a baby dying after 28 weeks of pregnancy but before or during delivery.

In this review, all quantitative observational studies reported on the relationship between IPV during pregnancy and the outcomes of interest were included. Meanwhile, the articles included in the present study had to meet the following criteria: (1) English language; (2) published in/after 1 January 2011; (3) raw data included. However, observational studies that did not report on original research (i.e., conference abstract, comment, editorial or letter, review) or just qualitative studies were excluded.

## 3. Methods

### 3.1. Search strategy

We searched four databases including EMBASE, Web of Science, PubMed, and CINAHL from 1 January 2011 to 31 August 2021. We planned to summarize contemporary literature through this review, thus, the included studies were limited to the past 10 years, considering that the birth outcomes may be influenced by environmental, behavioral, and sociodemographic factors (13–15). In addition, eligible articles were also attained by reviewing references. We used a combination of keywords and medical subject headings (MeSH) to search: intimate partner violence, partner abuse, spouse abuse, domestic abuse, battered women, preterm/premature birth/labor/delivery, low birth weight, and stillbirth. The detailed information is described in Supplementary Table 1.

This systematic review was conducted according to the Preferred Reporting Items for Systematic Reviews and Meta-Analyses (PRISMA) (16). We have registered this study in the International Prospective Register of Systematic Reviews (PROSPERO) system (Registration No.: CRD42021282936), and no similar reviews were listed in the PROSPERO database before registration.

### 3.2. Data extraction

Two independent reviewers (Guo and Wan) searched for articles that met the inclusion criteria, obtained potentially relevant articles through a preliminary screening of the titles and abstracts, and then read the full text of the literature and determined its eligibility through the exclusion criteria. For any discrepancies, a third reviewer (Ge) was consulted, and we would reach a consensus. Then, two reviewers (Guo and Wan) independently extracted the data from the eligible studies using a pre-prepared form. Extracted data were reviewed and verified by the reviewer (Ge). The form mainly included study characteristics (authors, year of publication, study design, sample size, and study location), type of IPV (physical, sexual, and emotional) and the abuse assessment tool, and the adverse birth outcomes (i.e., preterm birth, low birth weight, and stillbirth).

### 3.3. Assessment of study quality

We critically appraised all included studies using the Joanna Briggs Institute (JBI) checklist for cross-sectional studies, the JBI checklist for cohort studies, and the JBI checklist for case–control studies (17). Briefly, the checklist for cross-sectional study, cohort study, and case–control study includes a total of eight, 11, and 10 items, respectively. Each item is divided into three grades: 1 = Yes, 2 = No, 3 = Not applicable, with lower scores reflecting higher quality (ranging from 8 to 24 for cross-sectional studies, 11 to 33 for cohort studies, and 10 to 30 for case–control studies). Guo and Wan conducted quality assessments independently when extracting the data for meta-analysis. Any discrepancies were discussed and reached a consensus among the different reviewers.

### 3.4. Data synthesis and statistical analysis

We calculated the odds ratio (OR) with a corresponding 95% confidence interval (CI) for preterm birth, low birth weight, and stillbirth using a random-effect model, which is suitable where there is heterogeneity between included studies. Heterogeneity across studies was assessed using an *I*^2^ statistic accompanied by a chi-square *p*-value. Heterogeneity was assigned adjectives of low, moderate, and high to *I*^2^ values of 25, 50, and 75% (18). Publication bias was assessed using a funnel plot and Peter's test (19). We used leave-one-out sensitivity analysis to explore the influence of small sample size studies or low-quality studies. Meta-regression and subgroup analysis were performed to explore the source of heterogeneity (*I*^2^ ≥ 50%). The aforementioned analyses were based on the year of publication (as a continuous variable), development level (developed countries and developing countries), study quality (continuous), and measurement of IPV (self-report questionnaires and face-to-face interviews). All analyses were conducted in RStudio-4.0 using the meta package.

## 4. Results

### 4.1. Study and sample characteristics

**Figure 1:**
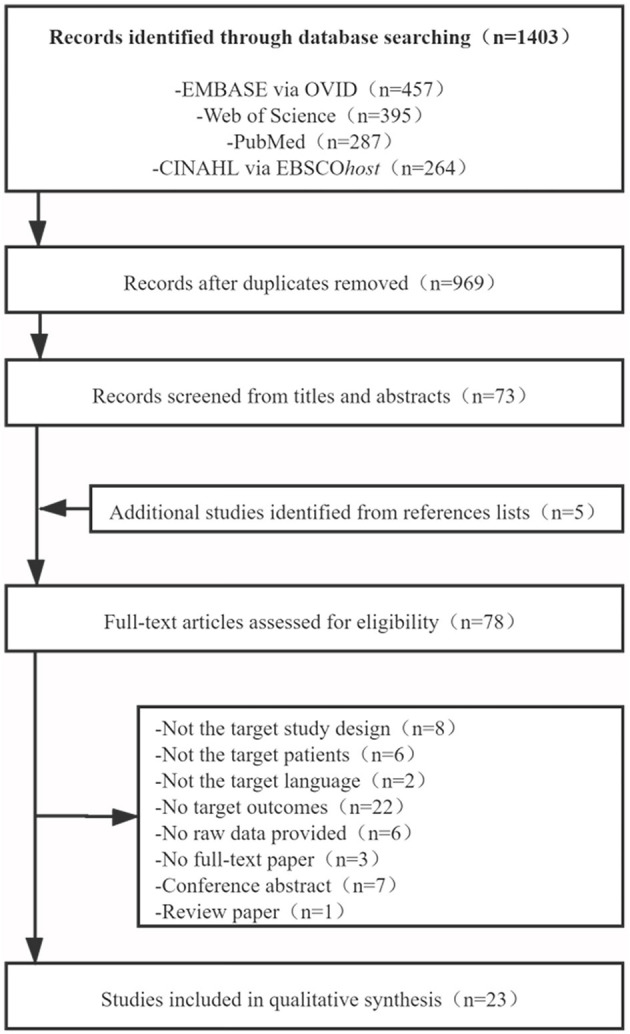
Flow diagram of study selection.

A total of 1,403 articles were identified through the four databases. After removing the duplicates and preliminary screening titles and abstracts, 73 studies were selected and read in full text to assess the eligibility along with five studies identified from the references lists. Ultimately, 23 studies were included, meeting the aforementioned inclusion and exclusion criteria, and providing the raw data that needed to be included in the meta-analysis (Figure 1).

Table 1 demonstrates the study design and sample characteristics of included studies. In total, 23 studies included a total of 42,089 middle-aged (ranging from 25 to 35) adult females. A total of 11 studies were conducted in Asia, eight studies were conducted in Africa, two studies were conducted in North America, one study was conducted in South America, and the last study was conducted in Europe. All eligible research articles are observational studies. Among the included studies, cross-sectional and cohort studies were each represented by 11 articles, while the remaining article was a case–control study. IPV was measured by self-report questionnaires in 18 studies (the most used was the modified *WHO Domestic Violence Questionnaire*, eight studies), and five studies were obtained through face-to-face interviews with doctors or nurses in hospitals. The majority of studies focused on physical IPV and single birth outcomes, mostly preterm as a birth outcome.

**Table 1:** Characteristics of studies included in the meta-analysis of IPV and birth outcomes.

**References**	**Study design**	**Sample size**	**Study location**	**Type of violence**	**Abuse assessment**	**Outcomes**
Abujilban et al. (20)	Cross-sectional	158	Jordan	Physical	Modified WHO Domestic Violence Questionnaire	Preterm birth
Abdollahi et al. (21)	Cohort	1,461	Iran	Physical	Modified WHO Domestic Violence Questionnaire	Low birth weight
Afkhamzadeh et al. (22)	Cohort	1,025	Iran	Physical, sexual, emotional	Modified WHO Domestic Violence Questionnaire	Preterm birth low birth weight
Al Shidhani et al. (23)	Cross-sectional	202	Oman	Physical, emotional	Arabic NorVold Domestic Abuse Questionnaire	Preterm birth low birth weight
Chen et al. (24)	Cohort	1,438	The United States	Physical, emotional	Hospital face-to-face interview	Preterm birth low birth weight
Elkhateeb et al. (25)	Cross-sectional	513	Egypt	Physical, sexual, emotional	Abuse Assessment Screen	Preterm birth
Eno et al. (26)	Case-control	200	Nigeria	Physical, sexual, emotional	Modified Abuse Assessment Screen	Preterm birth low birth weight
Fay et al. (27)	Cross-sectional	197	The United States	Physical, sexual	10-item Reproductive Coercion	Preterm birth low birth weight stillbirth
Gary et al. (28)	Cohort	1,500	India	Physical, sexual, emotional	Hospital face-to-face interview	Preterm birth stillbirth
Gebreslasie et al. (29)	Cross-sectional	647	Ethiopia	Physical, sexual, emotional	Hospital face-to-face interview	Stillbirth
Hassan et al. (30)	Cross-sectional	1,300	Iran	Physical, sexual, emotional	Abuse Assessment Screen Scale	Preterm birth
Hoang et al. (31)	Cohort	1,276	Vietnam	Physical, sexual, emotional	Modified WHO Domestic Violence Questionnaire	Preterm birth low birth weight stillbirth
Ibrahim et al. (32)	Cohort	1,857	Egypt	Physical, sexual, emotional	NorVold Domestic Abuse Questionnaire	Preterm birth low birth weight
Jain et al. (33)	Cohort	361	India	Physical, sexual, emotional	Violence assessment screen	Preterm birth low birth weight stillbirth
Khatoon et al. (34)	Cohort	270	India	Physical, sexual, emotional	Modified WHO domestic violence questionnaire	Preterm birth
Laelago et al. (35)	Cross-sectional	183	Ethiopia	Physical, sexual, emotional	Modified WHO domestic violence questionnaire	Preterm birth low birth weight stillbirth
Maciel et al. (36)	Cross-sectional	11,901	France	Physical	Self-assessment	Preterm birth low birth weight
Musa et al. (37)	Cross-sectional	603	Ethiopia	Physical, sexual, emotional	Modified WHO domestic violence questionnaire	Preterm birth Low birth weight
Navvabi-Rigi et al. (38)	Cohort	335	Iran	Physical, sexual	Hospital face-to-face interview	Low birth weight
Ramalingappa et al. (39)	Cohort	800	India	Physical, sexual, emotional	Hospital face-to-face interview	Preterm birth
Ramos et al. (40)	Cross-sectional	14,299	Colombia	Physical	Self-assessment	Preterm birth low birth weight
Sigalla et al. (41)	Cohort	1,112	Tanzania	Physical, sexual, emotional	Modified WHO domestic violence questionnaire	Preterm birth low birth weight
Yaya et al. (42)	Cross-sectional	451	Zimbabwe	Physical, sexual, emotional	Self-assessment	Preterm birth

### 4.2. Risk of bias in individual studies

**Figure 2:**
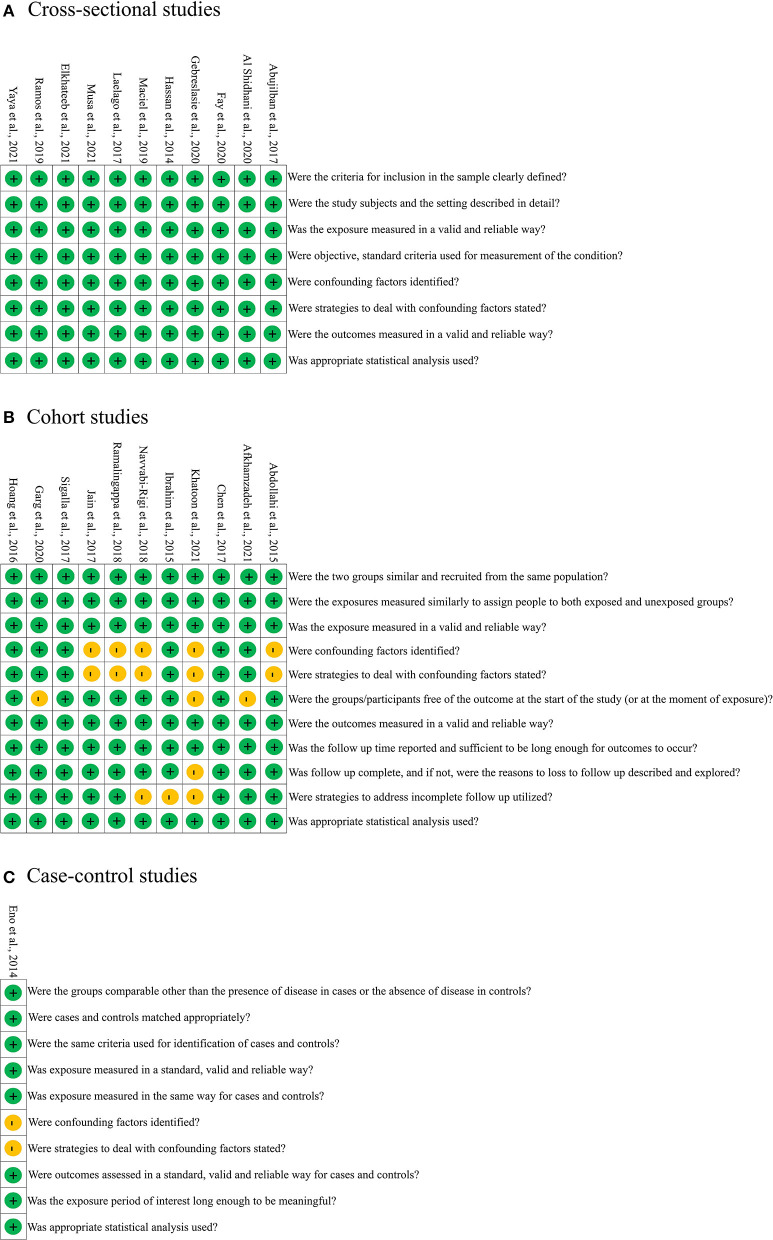
Risk of bias for the included cross-sectional **(A)**, cohort **(B)**, and case-control **(C)** studies.

As demonstrated in Figure 2A, all included cross-sectional studies indicated the criteria for inclusion of the sample in a clear way; the study subjects and settings were described in detail; the exposure was measured in a valid and reliable way; objective and standard criteria were used for the measurement of the condition; confounding factors were identified and dealt with appropriate strategies; and appropriate statistical analysis was used. As shown in Figure 2B, the included cohort studies indicated that the two groups were similar and recruited from the same population; the exposures were measured similarly to assign people to both exposed and unexposed groups; the outcomes were measured in a valid and reliable way; the follow-up time was reported and sufficient for outcomes to occur; and appropriate statistical analysis was used. Meanwhile, six of 11 studies accounted for all important confounding factors and dealt with them. Most of the included studies described the reasons to lose follow-up and utilized strategies to address incomplete follow-up. As can be seen from Figure 2C, Eno's study indicated the groups were comparable other than the presence of disease in cases or the absence of disease in controls; cases and controls were matched appropriately, and the same criteria were used for the identification of cases and controls; both exposure and outcome were measured in a standard, valid, and reliable way; the exposure period was long enough, and appropriate statistical analysis was used. This study did not indicate the confounding factors. Overall, the summary quality of all included studies was assessed as moderate to high.

### 4.3. Associations between IPV and risk of birth outcomes

**Figure 3:**
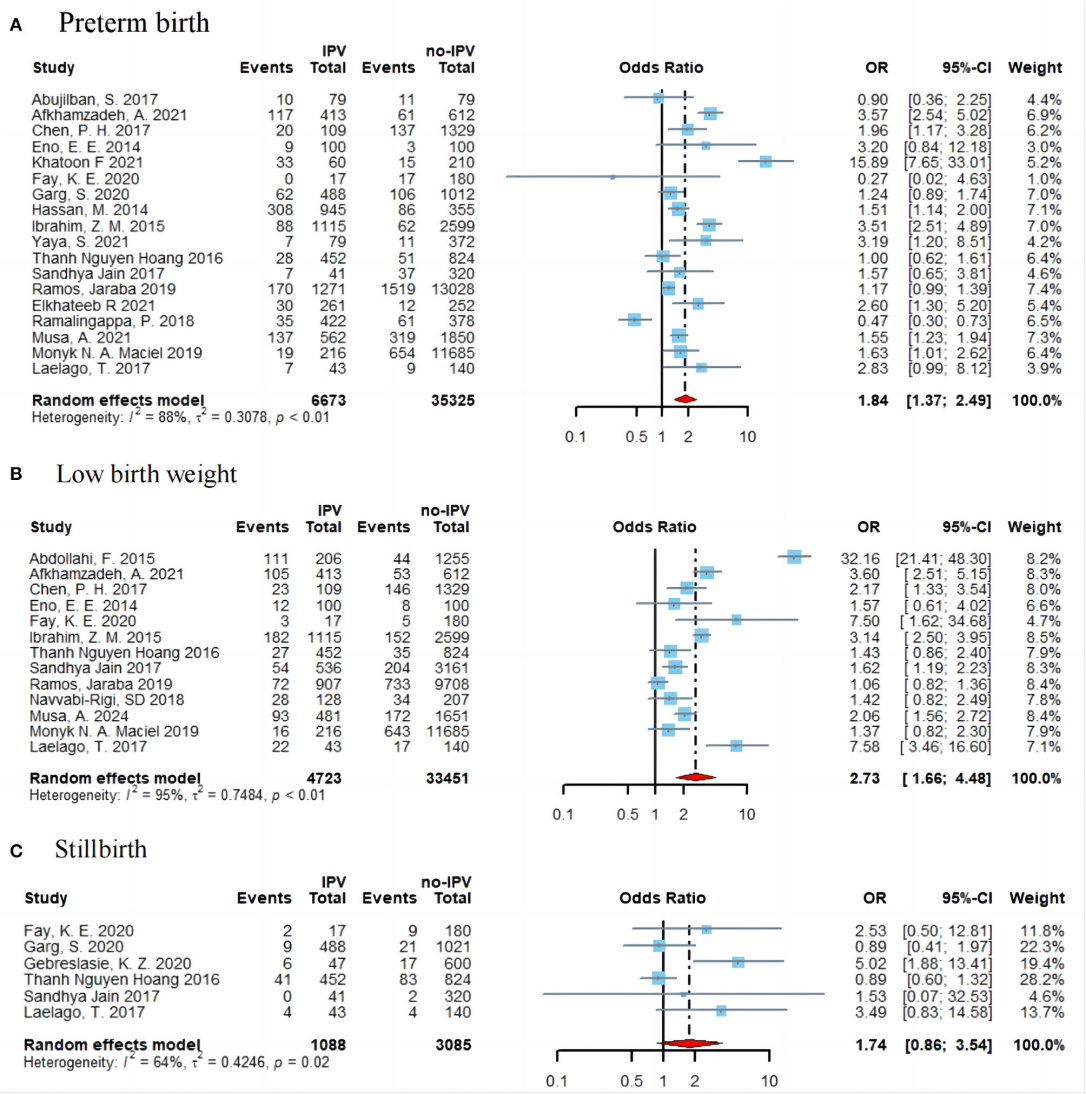
Forest plot of effect estimates for IPV in pregnancy and preterm birth **(A)**, low birth weight **(B)**, and stillbirth **(C)**.

As shown in Figure 3A, the association between IPV during pregnancy and preterm birth has been reported in 18 studies. Compared with women who did not experience IPV, victims of IPV during pregnancy were nearly twice as likely to give birth to a premature infant (OR = 1.84; 95% CI: 1.37–2.49), with high heterogeneity (*I*^2^ = 88%, *p* < 0.01). Based on 13 studies, the pooled OR for IPV associated with low birth weight was 2.73 (95% CI: 1.66–4.48) and heterogeneity is 95% (*p* < 0.01) (Figure 3B). As for stillbirth, six of the included studies showed that women who experienced IPV during pregnancy were more likely to experience it (OR = 1.74; 95% CI: 0.86–3.54; *I*^2^ = 64%) (Figure 3C).

### 4.4. Associations between specific IPV and risk of birth outcomes

**Figure 4:**
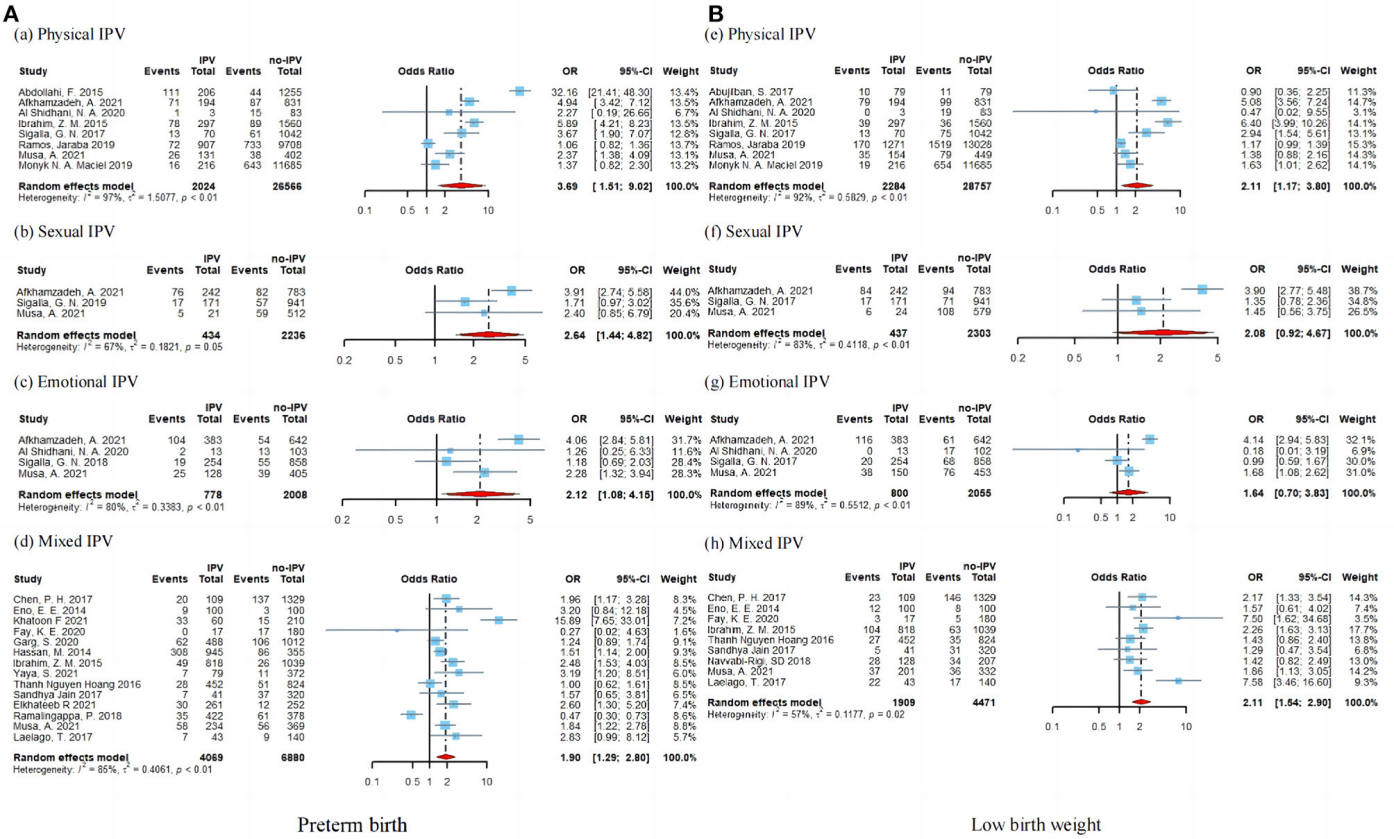
Subgroup analysis of preterm birth **(A)** and low birth weight **(B)** according to IPV type.

As shown in Figure 4, the association between preterm birth and low birth weight was still significant by specific IPV types. We did not explore the association between specific IPV types and stillbirth due to a small number of available studies. For the preterm birth (Figure 4A), significant associations were found in physical IPV (OR = 2.11; 95% CI: 1.17–3.80; *I*^2^ = 97%), sexual IPV (OR = 2.64; 95% CI: 1.44–4.82; *I*^2^ = 67%), emotional IPV (OR = 2.12; 95% CI 1.08–4.16; *I*^2^ = 80%), and mixed IPV (OR = 1.90; 95% CI: 1.29–2.80; *I*^2^ = 85%). For the low birth weight, the ORs for the association of the specific IPV (physical, sexual, emotional, and mixed IPV) were, respectively, 3.69 (95% CI: 1.51–9.02), 2.64 (95% CI: 1.44–4.82), 2.12 (95% CI: 1.08–4.15), and 2.11 (95% CI: 1.54–2.90) (Figure 4B).

### 4.5. Publication bias, meta-regression, and subgroup analyses

Visual inspection of the funnel plots for preterm birth, low birth weight, and stillbirth showed little publication bias (Supplementary Figure 1). Peter's test is 0.33 and 0.30 for preterm birth and low birth weight outcomes, respectively. Due to the small number of studies (< 10 studies), we were unable to conduct Peter's test for stillbirth outcomes. We did not significantly alter the pooled OR for preterm birth, low birth weight, and stillbirth in the leave-one-out sensitivity analysis (Supplementary Figure 2). We performed univariate meta-regression models and subgroup analyses to detect the possible sources of heterogeneity according to the year of publication, level of development, study quality, and measurement of IPV, but none of the above variables were found to be statistically significant (*p*-value > 0.05). The details are shown in Supplementary Figure 3.

## 5. Discussion

The aim of the present study is to synthesize the recent studies involving the association between IPV during pregnancy and adverse birth outcomes. At the global scale, all subtypes of IPV were associated with an increased risk of adverse birth outcomes, especially for physical IPV. To evaluate the robustness of our findings, we calculated the association in different subgroups, including the development level, study quality, and measurement of IPV.

Consistent with previous studies, we found that IPV is a risk factor that can lead to severe birth outcomes involving preterm birth, low birth weight, and stillbirth (43, 44). Donovan et al. (43) found that women exposed to IPV during pregnancy were more likely to deliver a premature or low birth weight infant than women who did not experience IPV. In addition, there is also a positive association between IPV during pregnancy and stillbirth (44). Although the mechanism of the associations between IPV and adverse birth outcomes was unclear, previous studies explored it from the following aspects. First, physical IPV can directly affect fetal growth through trauma (45), which may explain the stronger association between physical IPV and adverse birth outcomes in the subgroup analysis. One possible reason is that violence may lead to pregnancy complications, such as placental damage, uterine contractions, and premature rupture of membranes (45, 46) and then followed by preterm birth and low birth weight. Second, sexual violence can increase the risk of sexually transmitted diseases and genitourinary infections (47). Third, maltreatment can lead to anxiety and depression in pregnant women, on the one hand (48, 49), and increased behavioral risk factors associated with adverse birth outcomes, on the other hand, such as maternal smoking, alcohol abuse, or less weight gain during pregnancy (50, 51). Premature rupture of membranes, depression, smoking, drinking, and other factors mentioned above are all risk factors for adverse birth outcomes (13–15), especially for preterm birth and low birth weight. We obtained comparable results across different subgroups (e.g., development level or study quality) and meta-regression analyses, which suggested that the observed associations could not be modified by study-level characteristics.

This study evaluated the association between IPV during pregnancy and preterm birth, low birth weight, and stillbirth and showed that IPV during pregnancy can not only harm the pregnant woman herself but also adversely affect the birth outcomes, which can more intuitively present the severity of IPV during pregnancy, so as to alert the health decision-makers and healthcare providers to its attention. The proportion of eligible birth outcomes is also an indicator of a country's socioeconomic development. Therefore, based on the results of this study and reality, we are likely to put forward a proposal; women who experience IPV during pregnancy should be identified as early as possible and given care and support to avoid more serious physical and emotional damage and worse birth outcomes. Prenatal care can be an important entry point for identifying violence among pregnant women, as under normal circumstances most people receive at least one or more prenatal care visits. Paradoxically, prenatal care and health services specifically for IPV during pregnancy are not available in almost all countries. IPV raises many ethical and legal issues, and to prevent and intervene in IPV during pregnancy, we must collaborate between healthcare, political, and social justice systems.

This meta-analysis has several strengths including considering stillbirth as an adverse birth outcome in our study. Although prior meta-analyses existed, none included stillbirth as an interesting outcome (7, 43, 44, 52, 53). Second, having a large sample size and detailed reported subtypes of IPV enabled us to assess the association between specific IPV (i.e., physical IPV, sexual IPV, emotional IPV, and mixed IPV) and adverse birth outcomes. Furthermore, we did some subgroup analysis (e.g., publication year, study location, and study quality) to avoid a spurious association. Our study has some limitations. First, the heterogeneity was high in our meta-analysis. This may be attributed to the cultural differences across countries. In some low-income countries (e.g., Ethiopia, Iran, and Tanzania), 81% of female victims reported that IPV is justified (54, 55); however, in some countries, the victims do not think so, such as Israel (56). Second, birth outcomes may be influenced by some behavior (e.g., smoking), maternal (e.g., overweight and obesity), and environmental (e.g., high temperature) factors (57–59). However, existing studies were not able to provide some detailed behavioral, maternal, and environmental information. Thus, it is difficult for us to control these potential confounders in our study.

## 6. Conclusion

This meta-analysis summarized all literature on the association between IPV during pregnancy and adverse birth outcomes in the last decade. Women who experienced IPV during pregnancy had a higher risk of adverse birth outcomes when compared to women who have not been exposed to IPV during pregnancy. All specific IPV, including physical, sexual, emotional, and mixed, were associated with adverse birth outcomes. In the future, more research on IPV during pregnancy is warranted and there is also an urgent need for greater action to prevent or intervene in IPV during pregnancy.

## Data availability statement

The original contributions presented in the study are included in the article/Supplementary material, further inquiries can be directed to the corresponding authors.

## Author contributions

FG and JZ were responsible for the study's concept and design. CG and MW independently extracted the data. FG reviewed and verified extracted data. CG, MW, PW, and FG cleaned the data and performed the analyses. CG, MW, YW, MT-P, and FG drafted the article. All authors contributed to the interpretation of data. All authors read and approved the final manuscript.
