# Supplementary material for: Associations between intimate partner violence and adverse birth outcomes during pregnancy: a systematic review and meta-analysis
Source: Front Med (Lausanne). 2023 May 17;10:1140787. doi: 10.3389/fmed.2023.1140787 (PMC10230039; doi:10.3389/fmed.2023.1140787)
Supplement: Supplementary file 1 [file Data_Sheet_1.docx]

Supplementary Material

Associations between intimate partner violence and adverse birth outcomes during pregnancy: a systematic review and meta-analysis

Cancan Guo^1†^, Mengtong Wan^2†^, Yue Wang^3,4^, Peijie Wang^5^, Marissa Wan Lynn Tousey-Pfarrer^4^, Haoyang Liu^1^, Liangming Yu^1^, Lingqi Jian^1^, Mengting Zhang^1^, Ziqi Yang^1^, Fenfen Ge^3,4*‡^,Jun Zhang^3*‡^

^1^ West China School of Medicine, Sichuan University, Chengdu, China

^2^ School of Medicine, Shanghai Jiao Tong University, Shanghai, China

^3^ Mental Health Center, West China Hospital, Sichuan University, Chengdu, China

^4^ Center of Public Health Sciences, Faculty of Medicine, University of Iceland, Reykjavík, Iceland

^5^ School of Education, Tianjin University, Tianjin, China

^†^ These authors have contributed equally to this work and shared the first authorship.

^‡^ These authors have contributed equally to this work.

*Correspondence

Jun Zhang

zh2000jun2000@163.com

Fenfen Ge

fenfenge@hi.is

**Supplementary Tables**

**sTable 1** Search strategy

| EMBASE | Embase <1974 to 2021 September 09>  1 exp premature labor/ 49222  2 exp stillbirth/ 19281  3 low birth weight/ 38136  4 pregnancy outcome/ 66562  5 premature labor.ab. or premature labor.ti. or preterm labor.ab. or preterm labor.ti. or premature birth.ab. or premature birth.ti. or premature delivery.ab. or premature delivery.ti. or preterm birth.ab. or preterm birth.ti. or preterm delivery.ab. or preterm delivery.ti. or stillbirth.ab. or stillbirth.ti. or low birth weight.ab. or low birth weight.ti. or pregnancy outcomes.ab. or pregnancy outcomes.ti. or birth outcomes.ab. or birth outcomes.ti. 116413  6 partner violence/ 14199  7 domestic violence/ 9855  8 battered woman/ 3287  9 partner violence.ab. or partner violence.ti. or intimate partner violence.ab. or intimate partner violence.ti. or spouse abuse.ab. or spouse abuse.ti. or domestic violence.ab. or domestic violence.ti. or battered women.ab. or battered women.ti. 17625  10 1 or 2 or 3 or 4 or 5 185580  11 6 or 7 or 8 or 9 26668  12 10 and 11 649  13 limit 12 to last 10 years 457 | 457 |
| --- | --- | --- |
| Web of Science | (((((((((((TS=(“premature birth”)) OR TS=(“preterm birth”)) OR TS=(“premature labor”)) OR TS=(“preterm labor”)) OR TS=(“premature delivery”)) OR TS=(“preterm delivery”)) OR TS=(“stillbirth”)) OR TS=(“low birth weight”)) OR TS=(“pregnancy outcomes”)) OR TS=(“birth outcomes”)) AND (((((TS=(“partner abuse”)) OR TS=(“intimate partner violence”)) OR TS=(“spouse abuse”)) OR TS=(“domestic violence”)) OR TS=(“battered women”))) AND DOP=(2011-09-10/2021-09-10) | 395 |
| PubMed | (((((((((partner abuse[MeSH Terms]) OR (spouse abuse[MeSH Terms])) OR (domestic violence[MeSH Terms])) OR (battered women[MeSH Terms])) OR (partner abuse[Title/Abstract])) OR (intimate partner violence[Title/Abstract])) OR (spouse abuse[Title/Abstract])) OR (domestic violence[Title/Abstract])) OR (battered women[Title/Abstract]) AND (y_10[Filter])) AND ((((((((((((((((premature birth[MeSH Terms]) OR (preterm birth[MeSH Terms])) OR (premature labor[MeSH Terms])) OR (preterm labor[MeSH Terms])) OR (stillbirth[MeSH Terms])) OR (infants, low birth weight[MeSH Terms])) OR (pregnancy outcomes[MeSH Terms])) OR (premature birth[Title/Abstract])) OR (preterm birth[Title/Abstract])) OR (premature labor[Title/Abstract])) OR (preterm labor[Title/Abstract])) OR (premature delivery[Title/Abstract])) OR (preterm delivery[Title/Abstract])) OR (low birth weight[Title/Abstract])) OR (pregnancy outcomes[Title/Abstract])) OR (birth outcomes[Title/Abstract]) AND (y_10[Filter])) | 287 |
| CINAHL | ((MH "Intimate Partner Violence") OR (MH "Domestic Violence") OR (MH "Battered Women") OR (TI ( partner abuse OR partner violence OR intimate partner violence OR spouse violence OR domestic violence OR battered women ) OR AB ( partner abuse OR partner violence OR intimate partner violence OR spouse violence OR domestic violence OR battered women ) )) AND ((MH "Childbirth, Premature") OR (MH "Perinatal Death") OR (MH "Pregnancy Outcomes") OR (MH "Infant, Low Birth Weight") OR (TI ( premature birth OR preterm birth OR premature labor OR preterm labor OR premature delivery OR preterm delivery ) OR AB ( premature birth OR preterm birth OR premature labor OR preterm labor OR premature delivery OR preterm delivery ) OR TI ( stillbirth or still birth or stillborn or still born or intrauterine death or perinatal death or dead baby in utero ) OR AB ( stillbirth or still birth or stillborn or still born or intrauterine death or perinatal death or dead baby in utero ) OR TI low birth weight OR AB low birth weight OR TI pregnancy outcomes OR AB pregnancy outcomes OR TI birth outcomes OR AB birth outcomes))  Limiters - Published Date: 20110101-20211231 | 264 |

**Supplementary Figures**

**(A)** Preterm birth


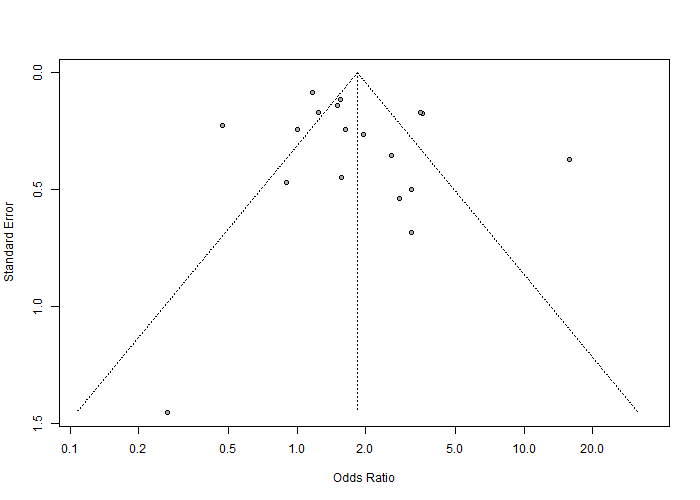


**(B)** Low birth weight


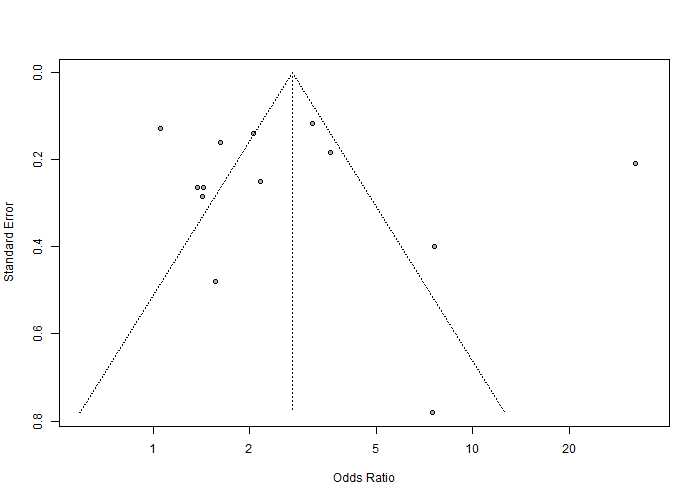


**(C)** Stillbirth


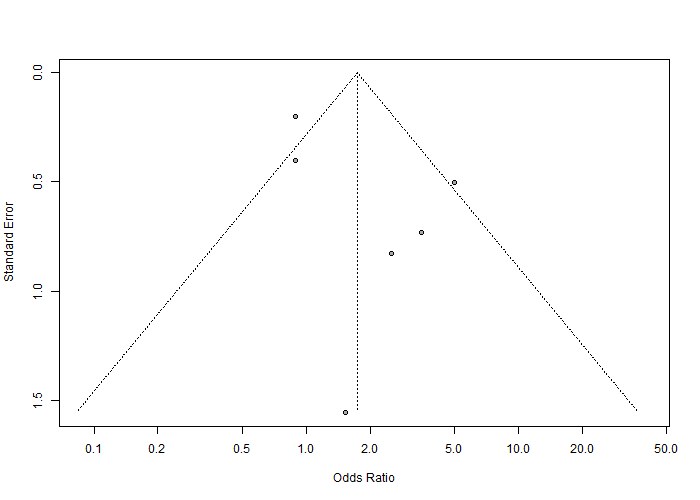


**sFigure 1** Qualitative detection of publication bias via 3 funnel plots

**(A)** Preterm birth


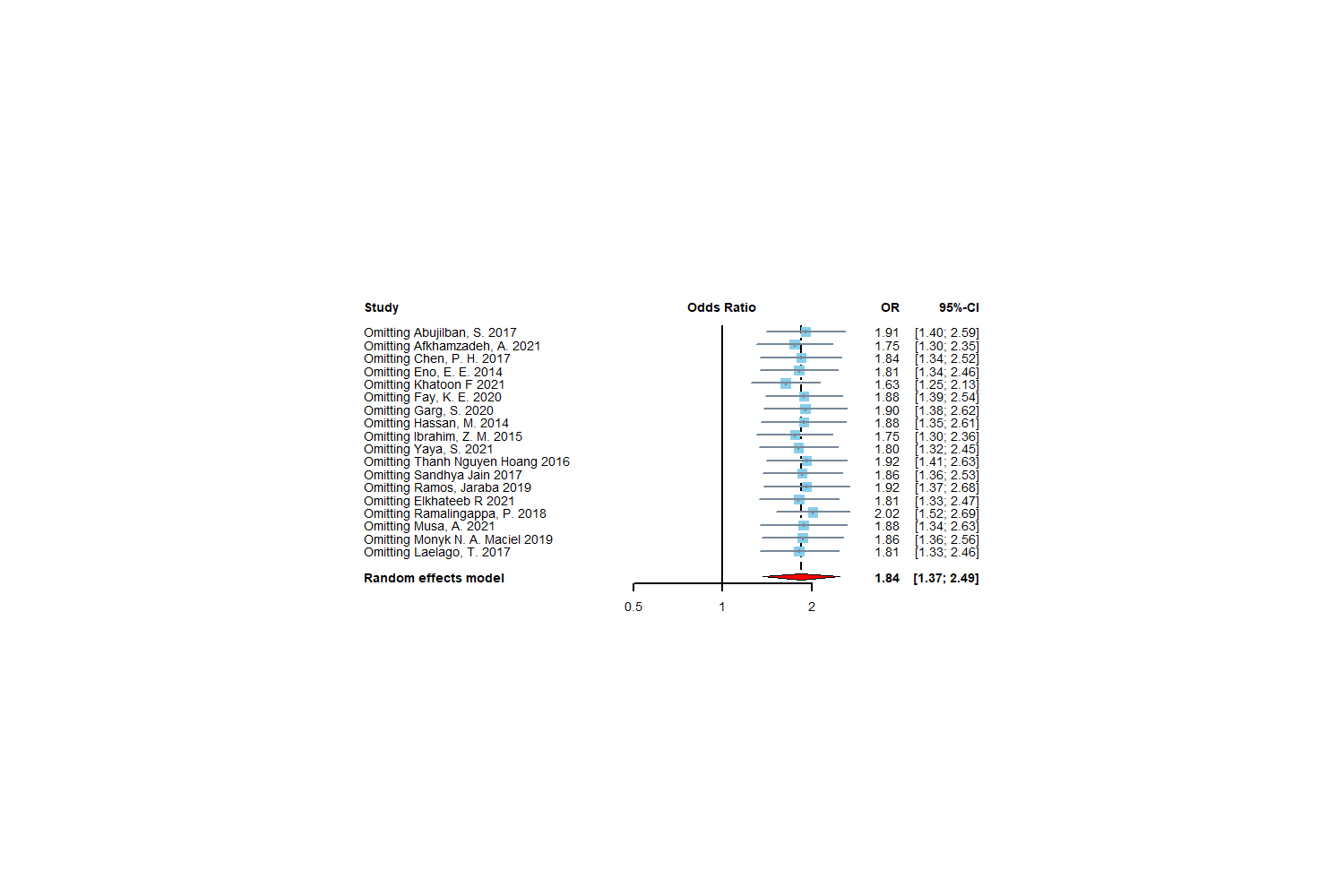


**(B)** Low birth weight


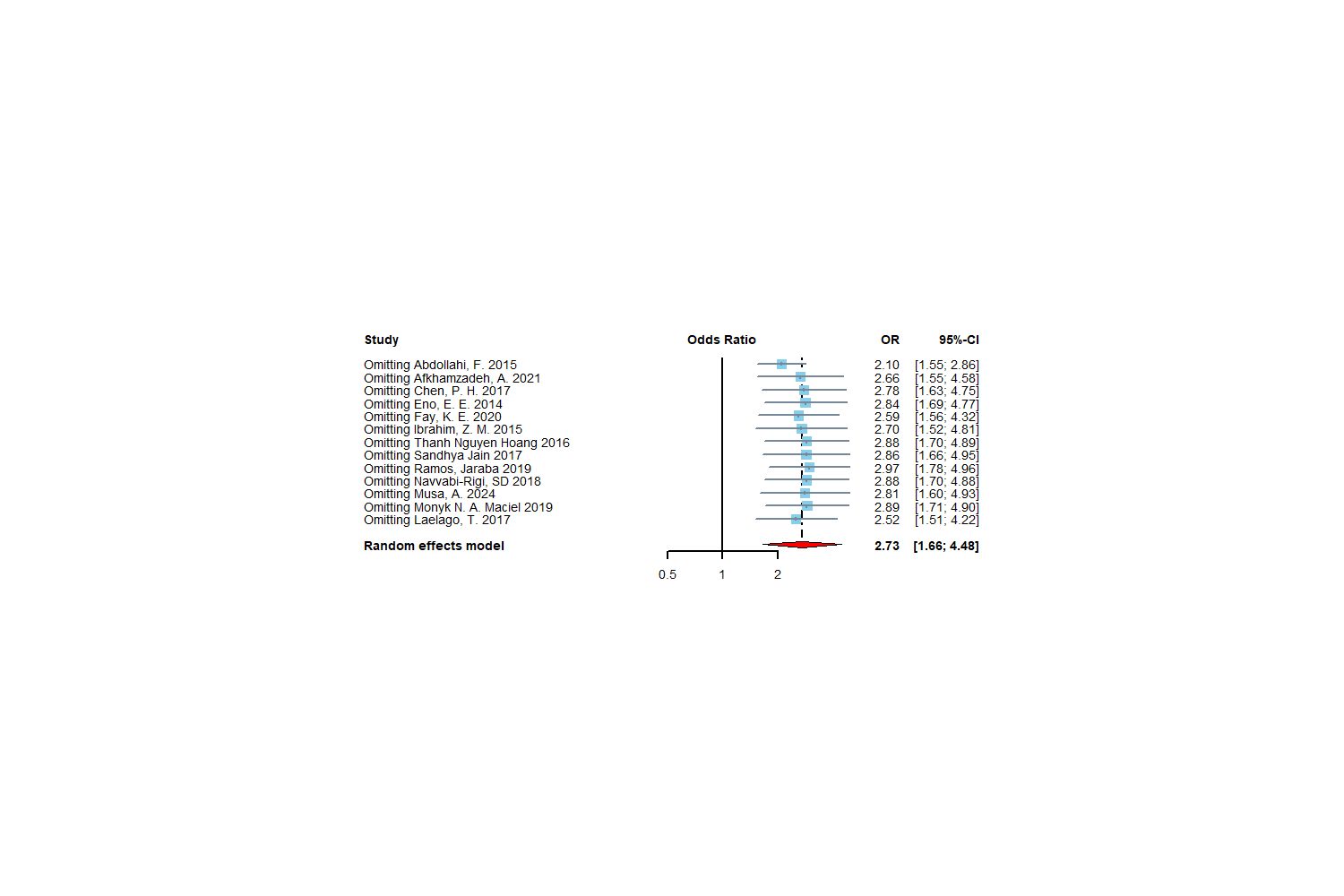


**(C)** Stillbirth


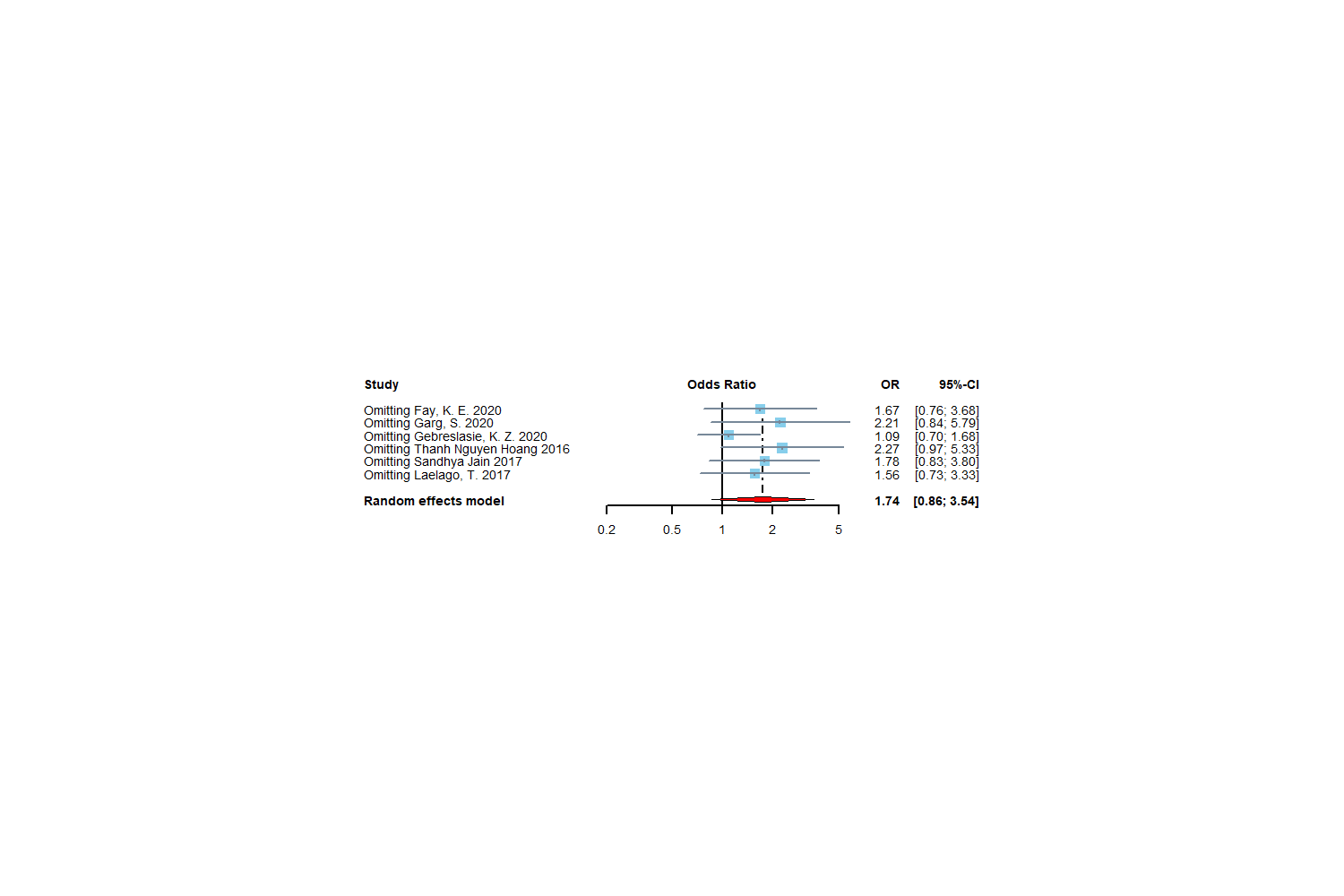


**sFigure 2** Forest plot of leave-one-out

**(A) Preterm birth**


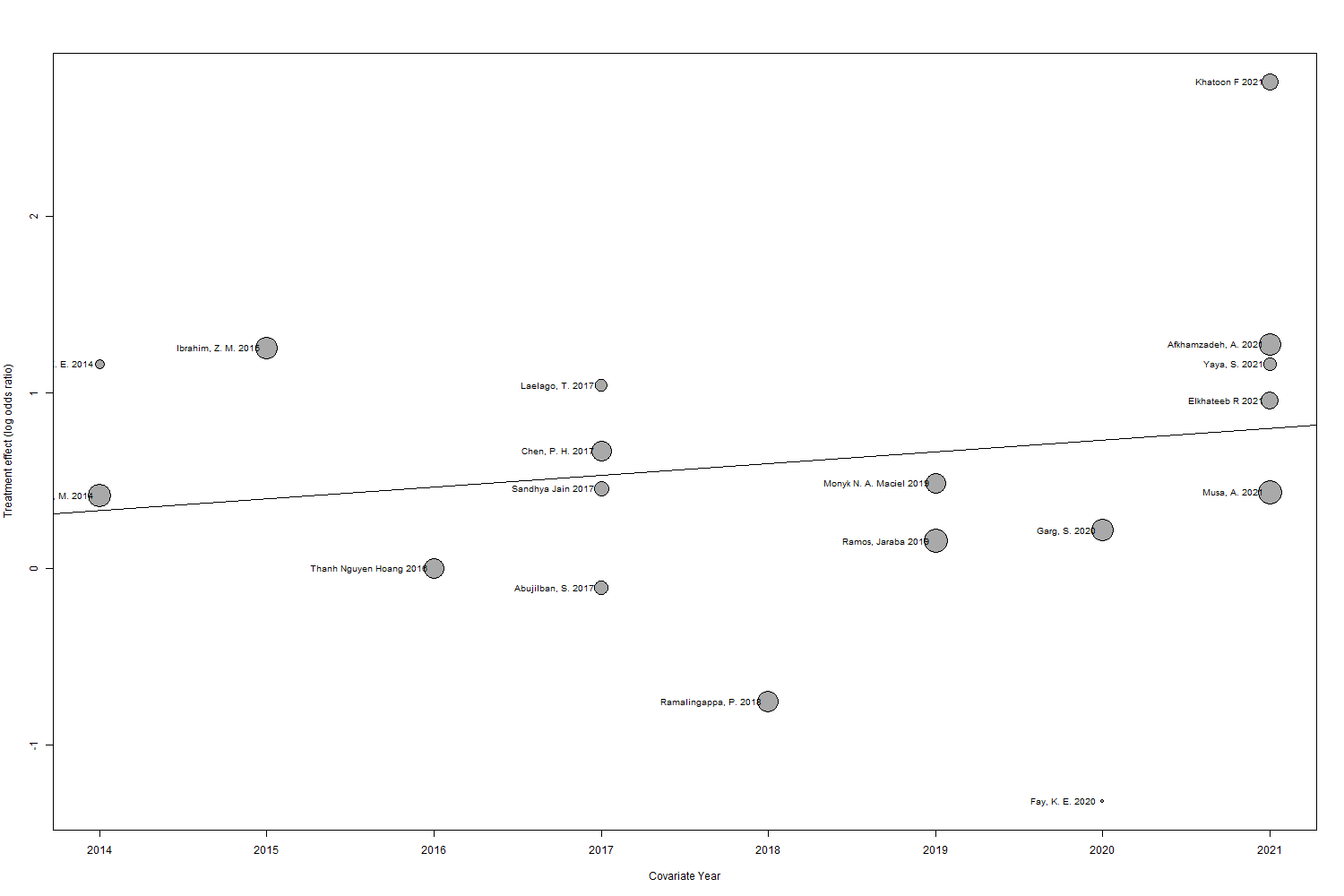


(a) Publication year


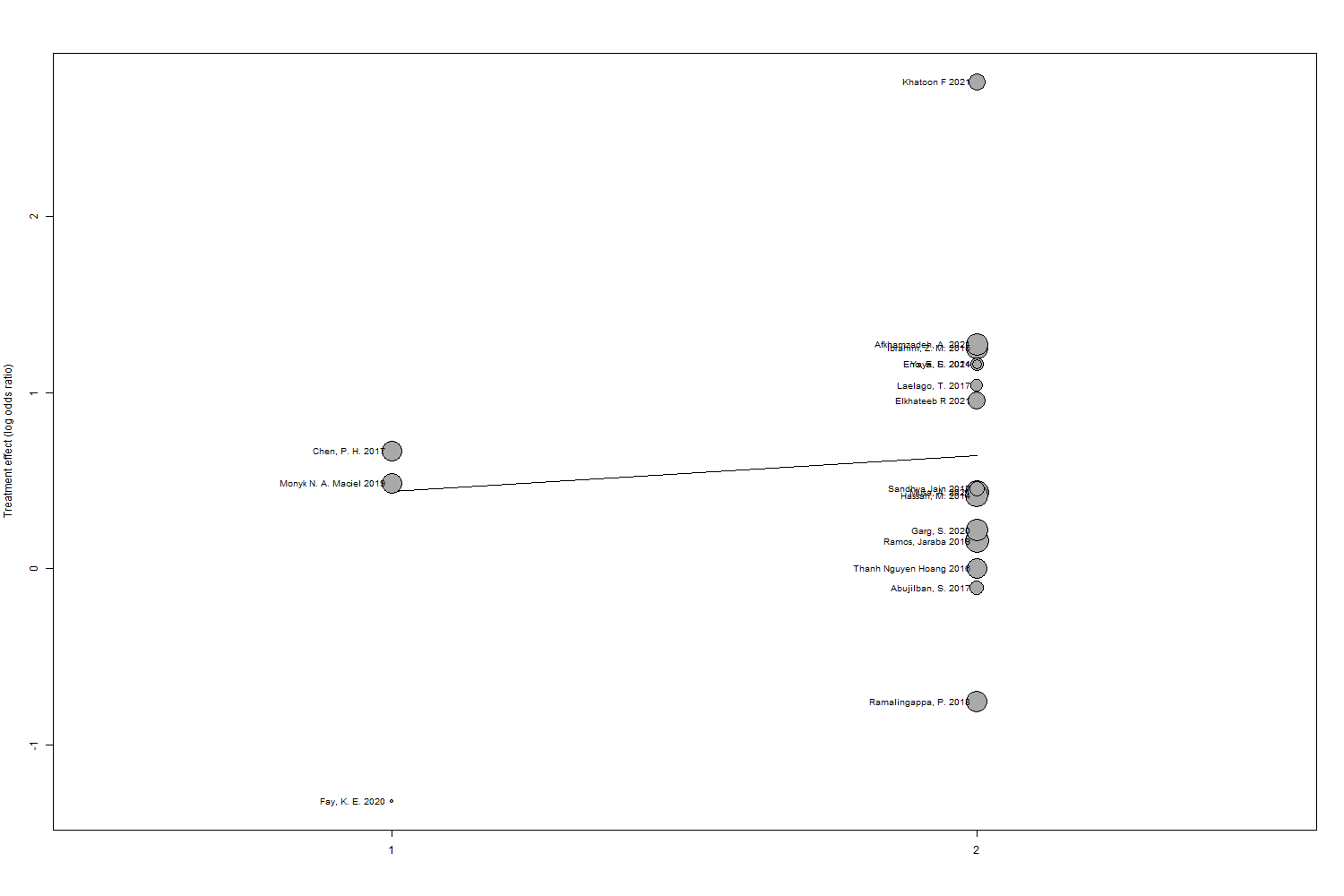


Developed countries

Developing countries

(b) Development level


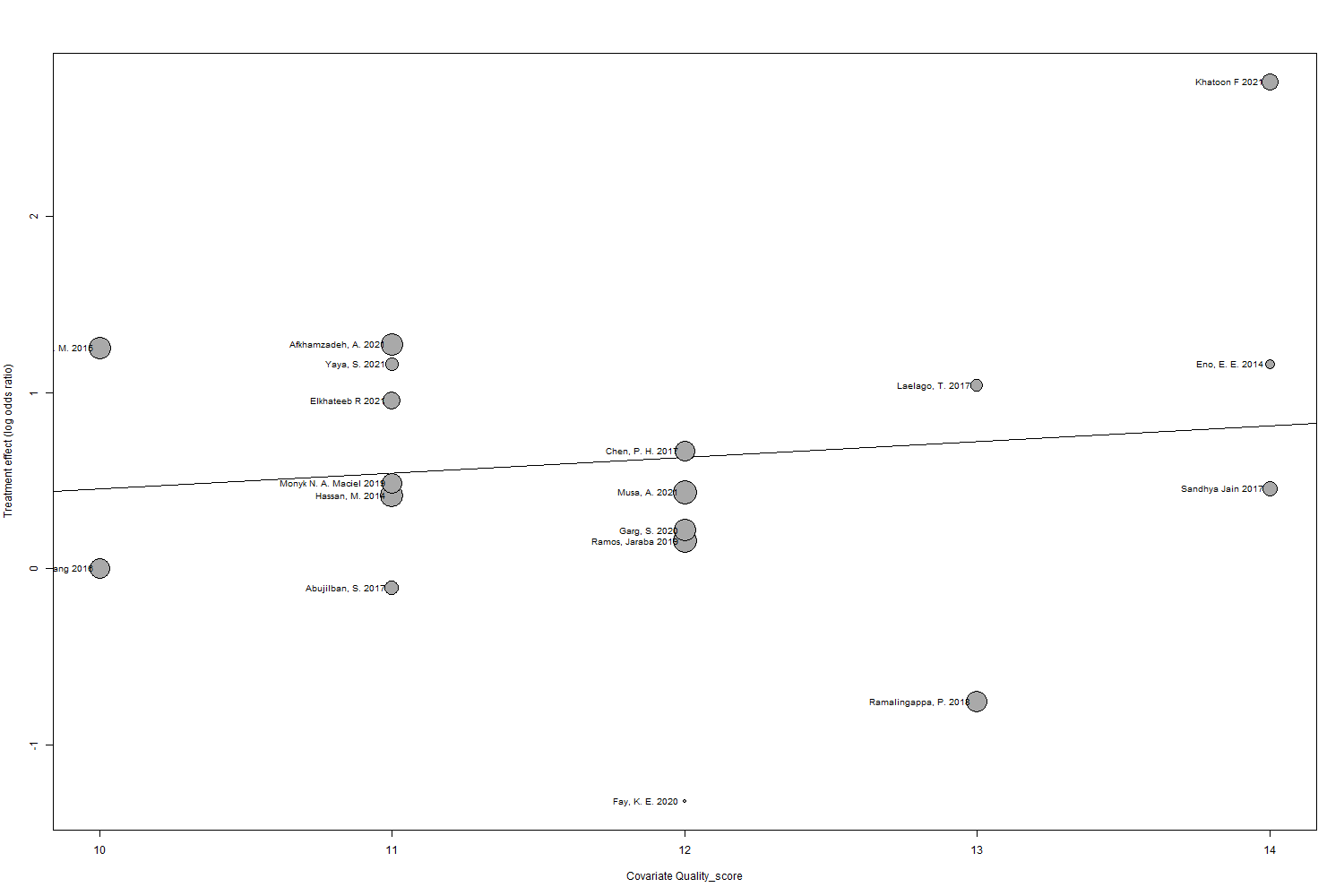


(c) Study quality


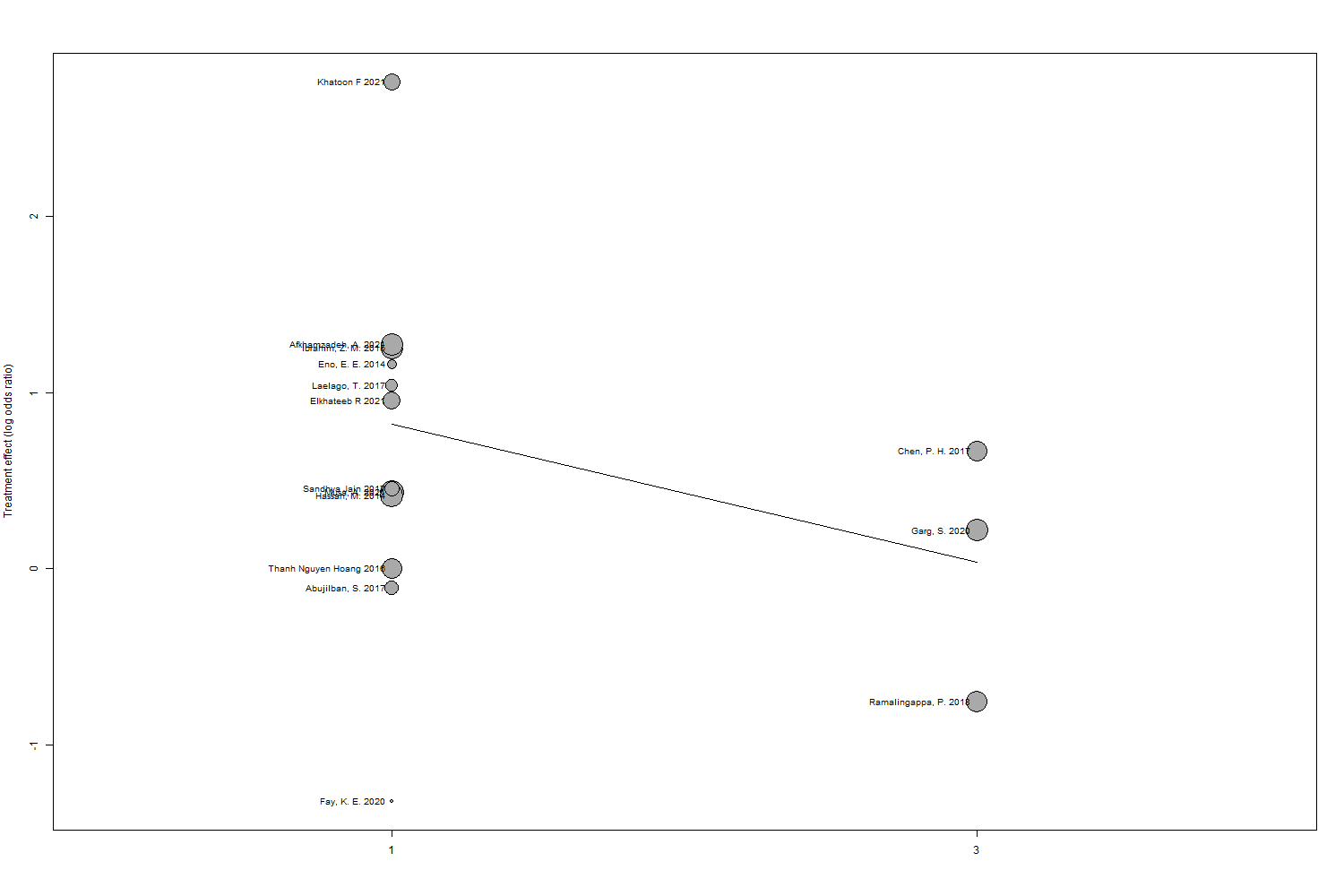


Self-report questionnaire

Face-to-face interview

(d) Assessment tool

**(B) Low birth weight**


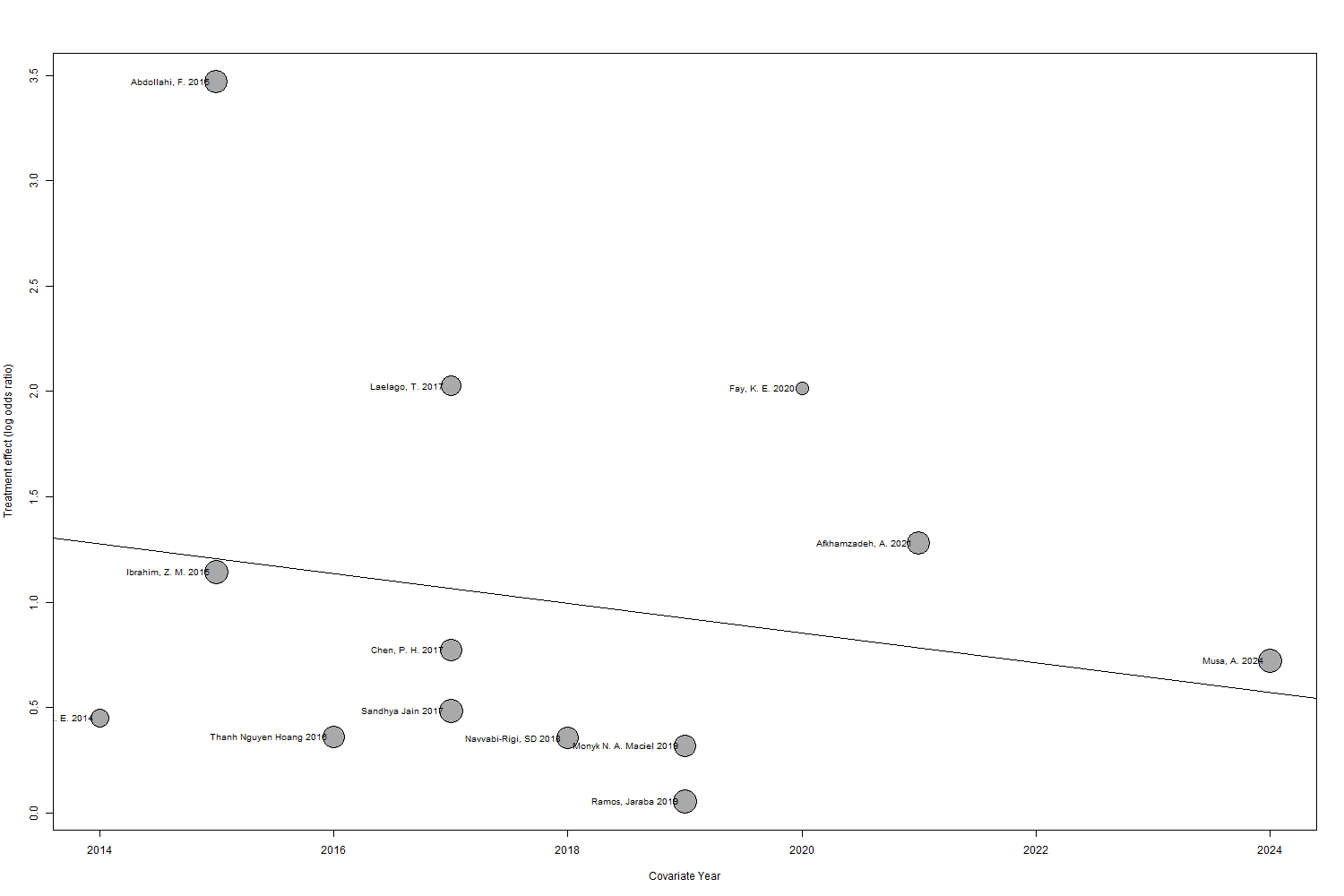


(a) Publication year


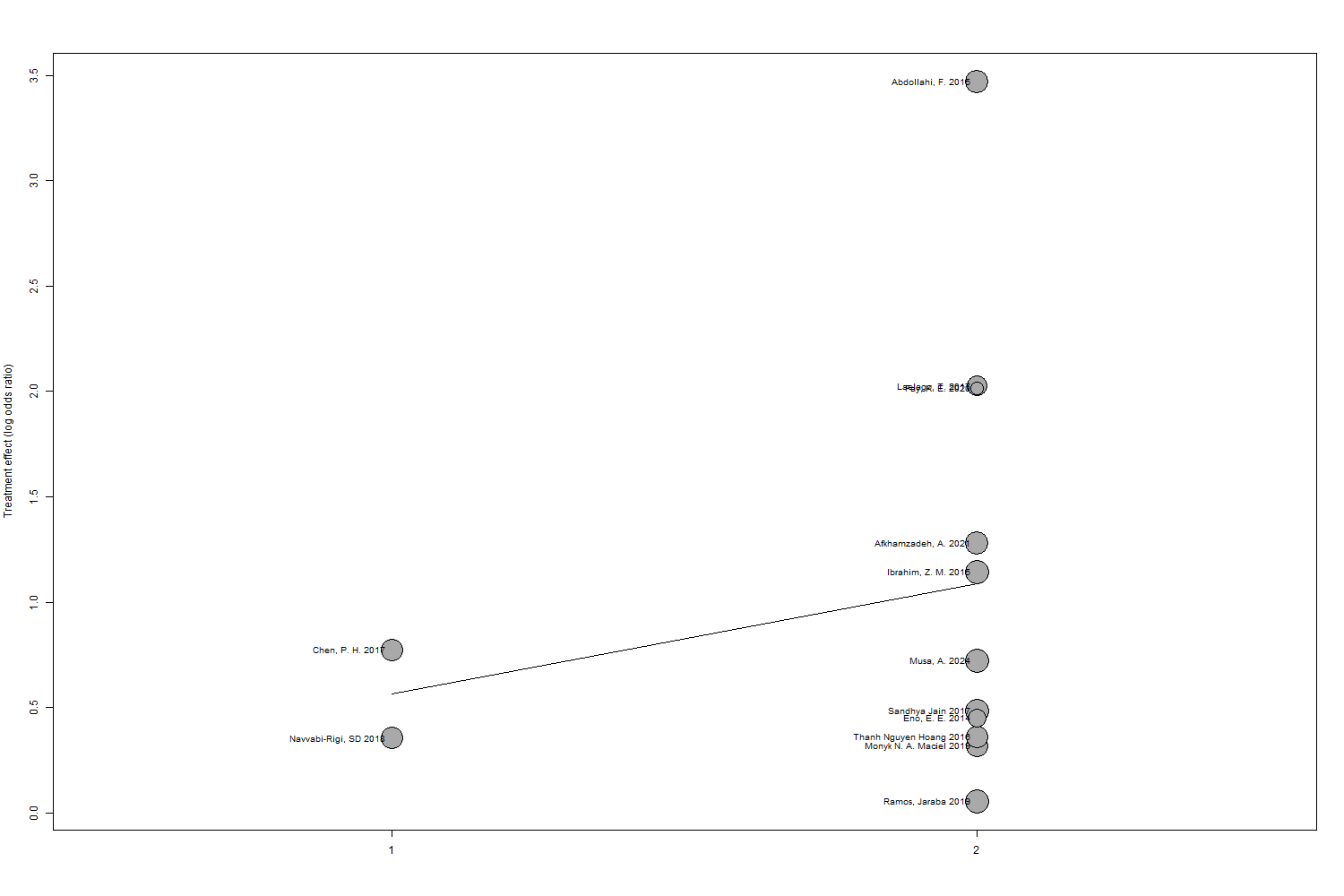


Developed countries

Developing countries

(b) Development level


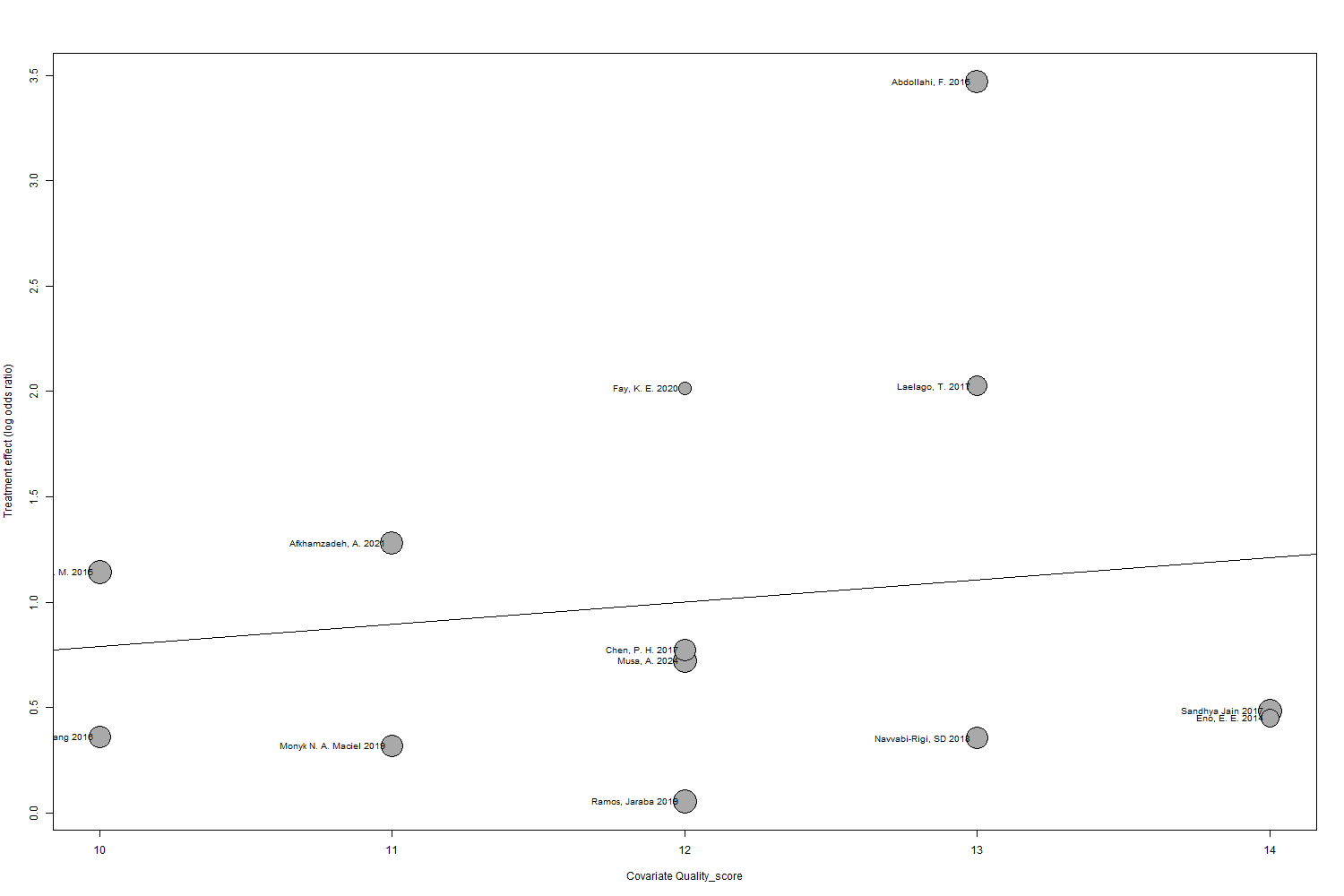


(c) Study quality


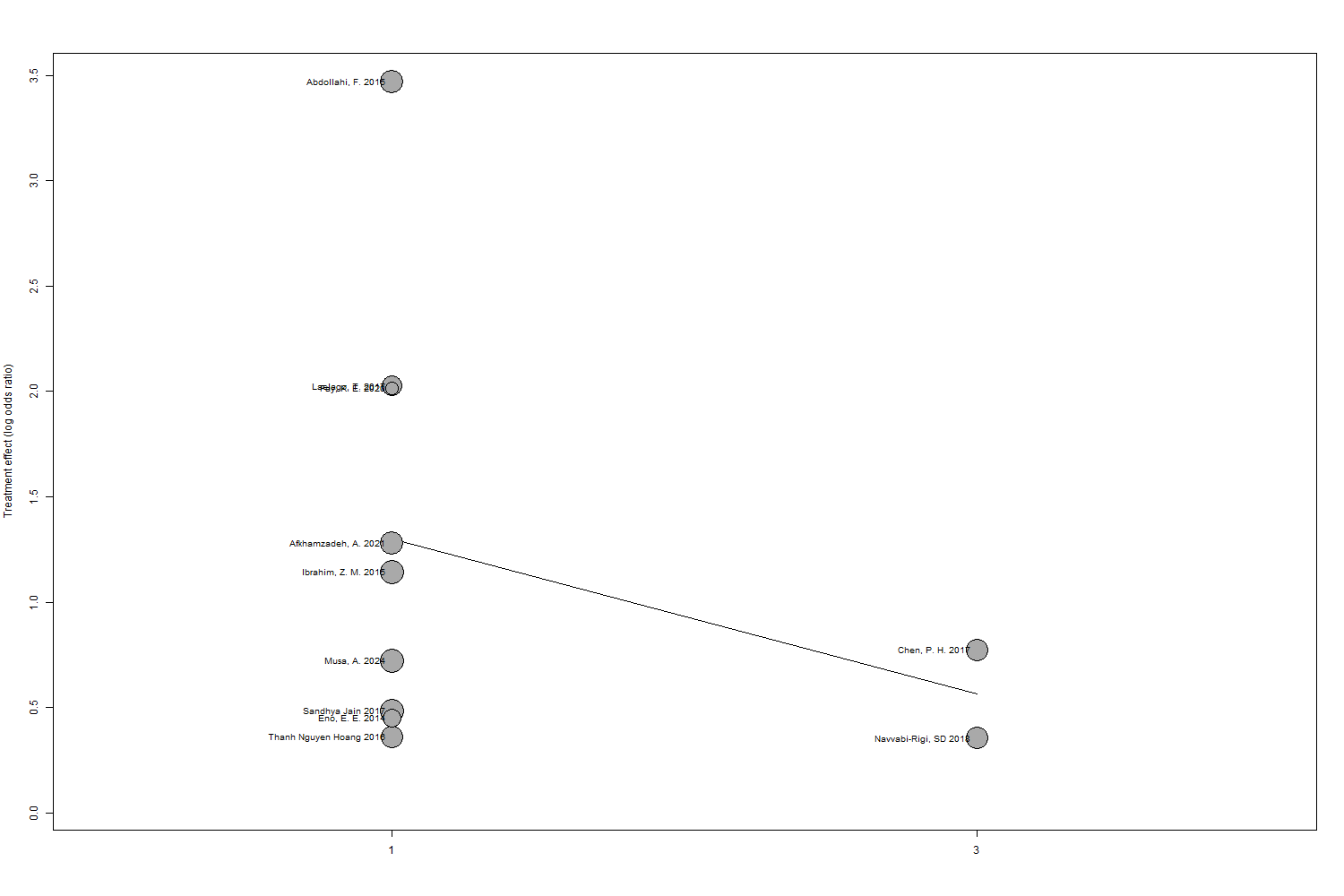


Self-report questionnaire

Face-to-face interview

(d) Assessment tool

**(C) Stillbirth**


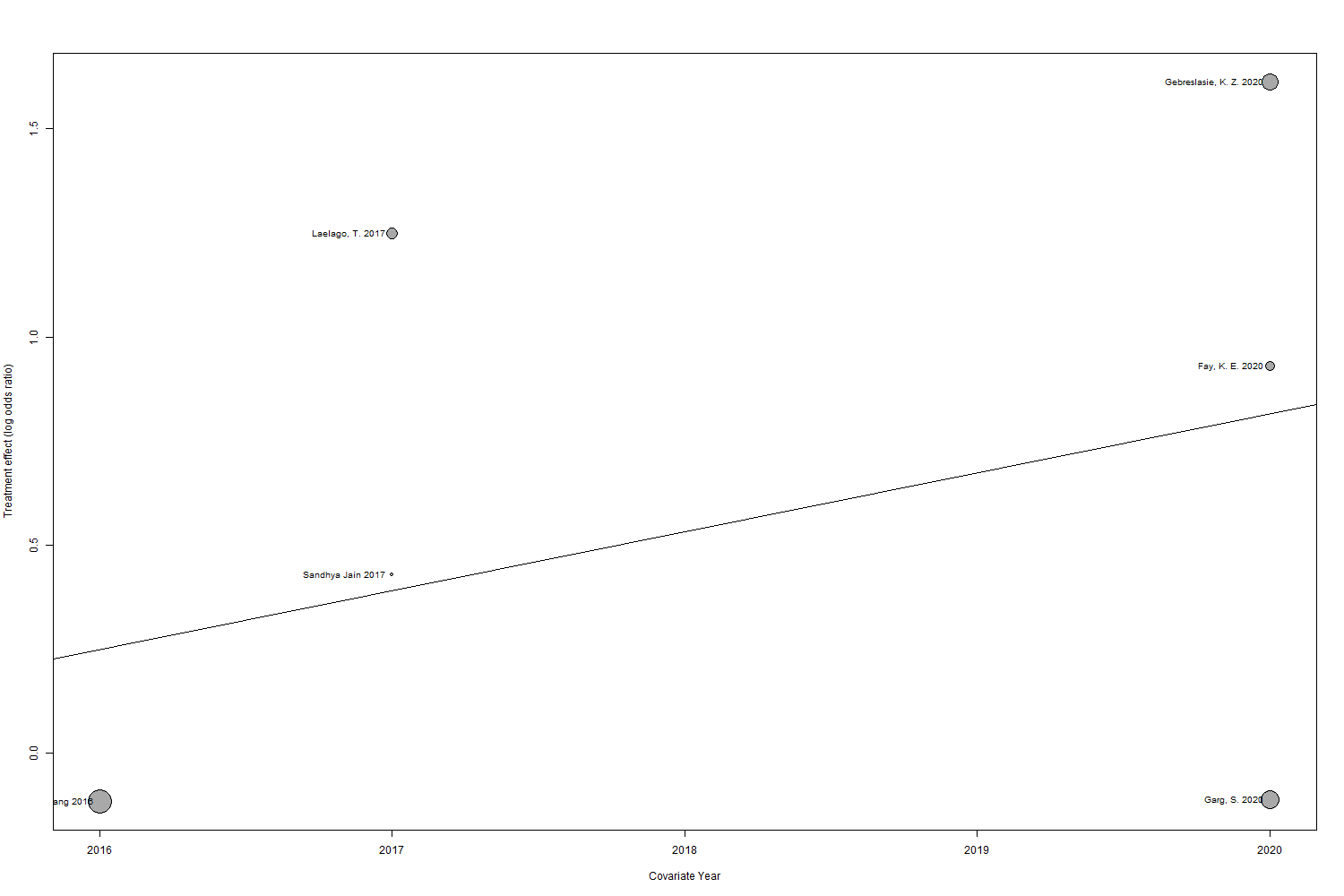


(a) Publication year


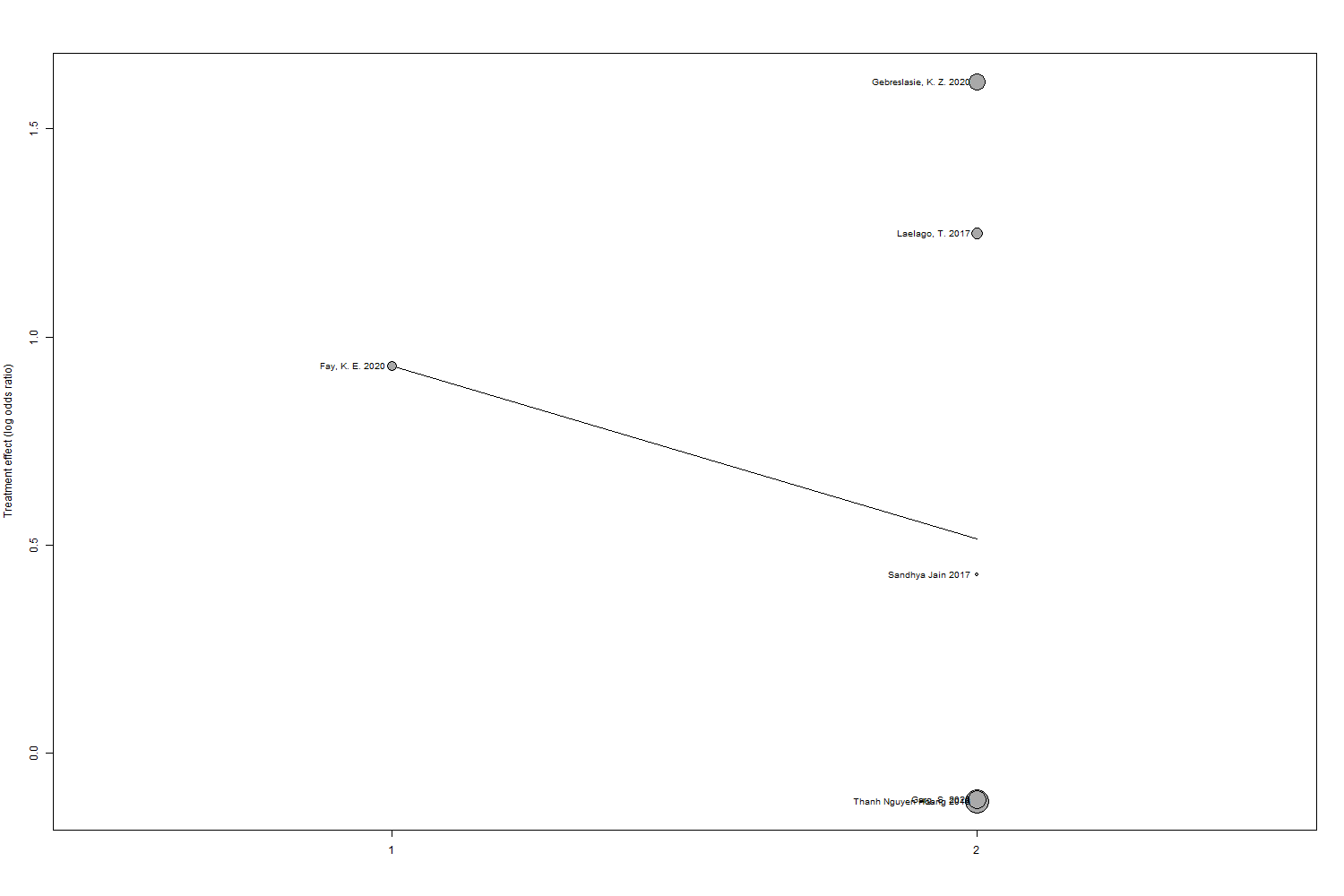


Developed countries

Developing countries

(b) Development level


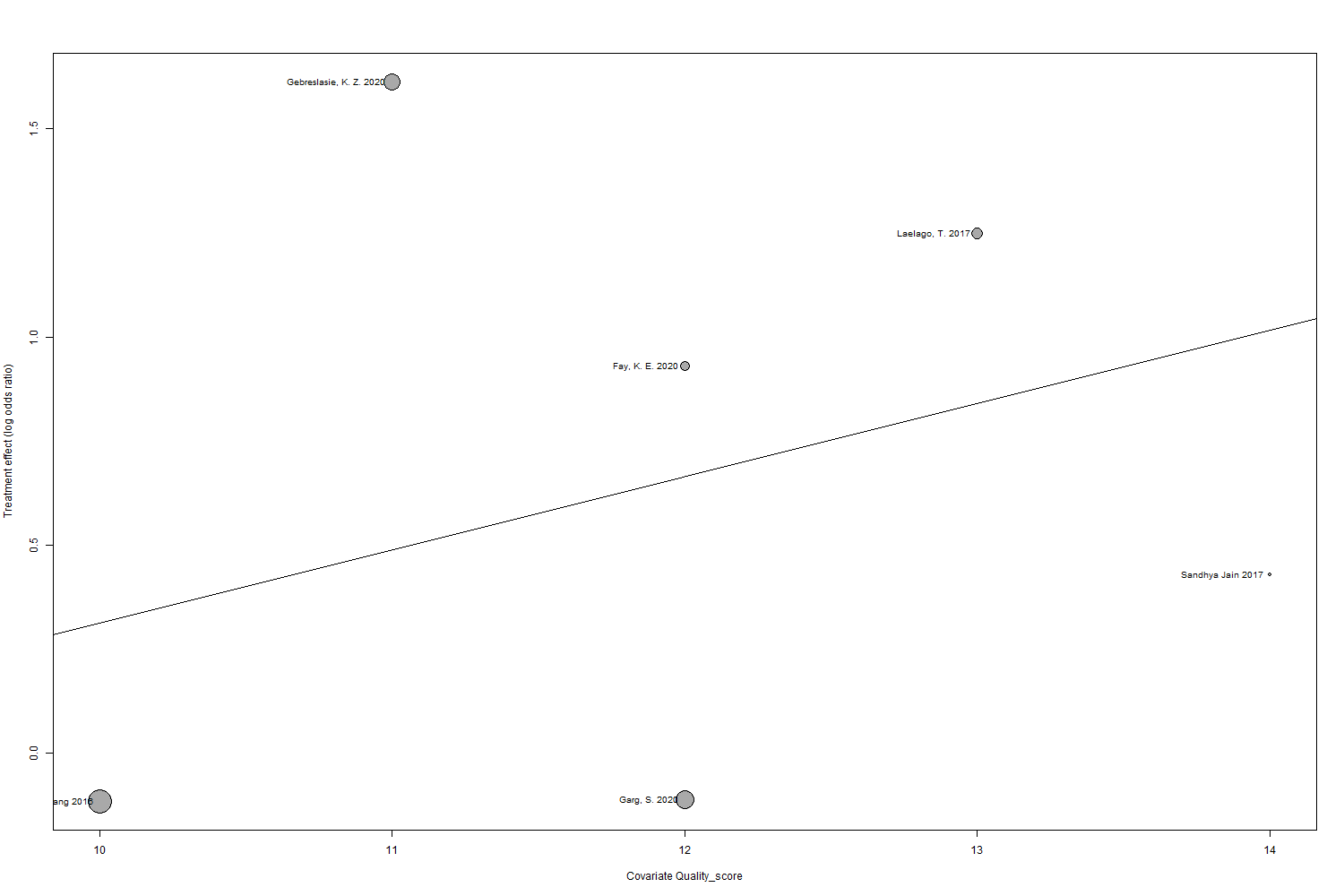


(c) Study quality


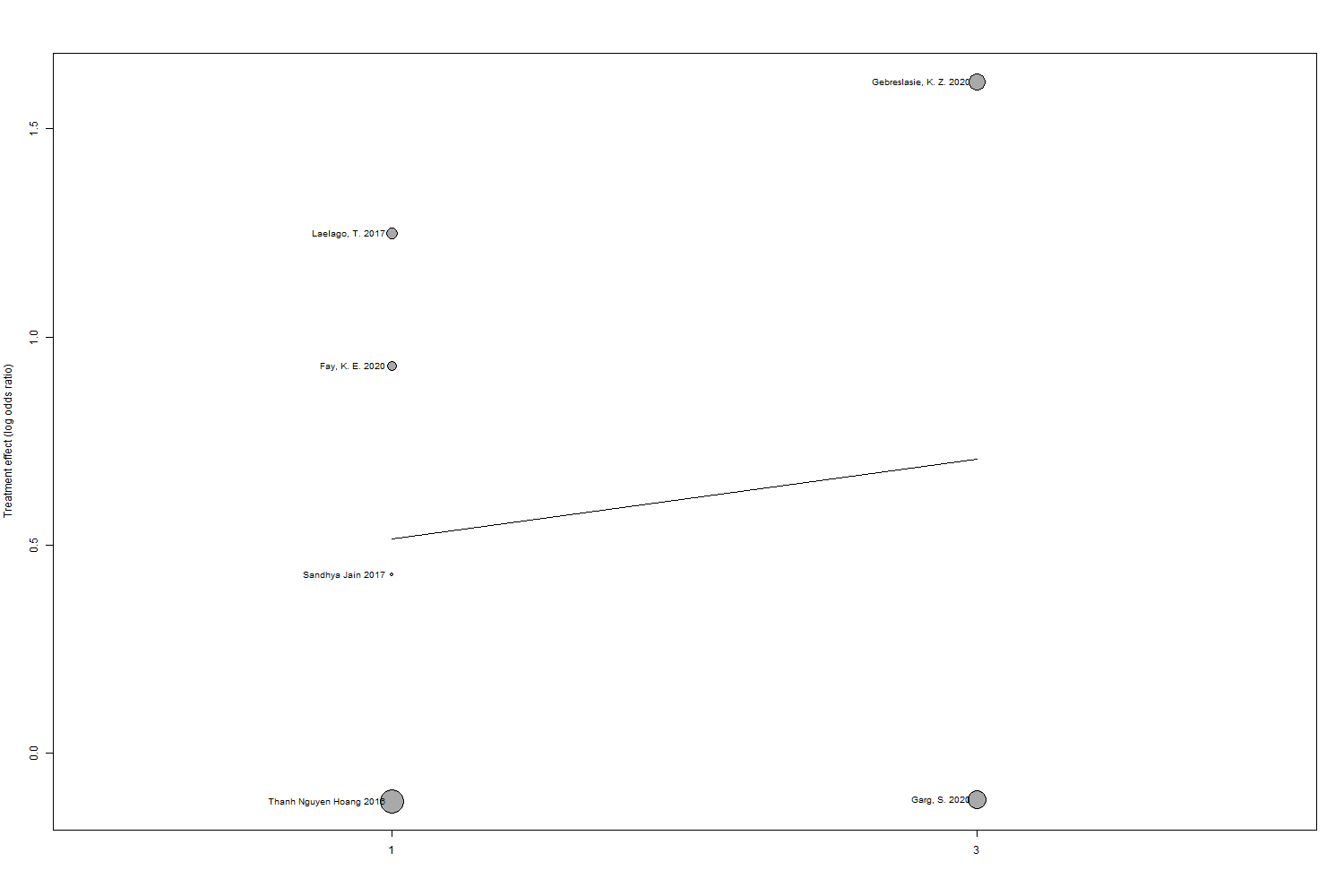


Self-report questionnaire

Face-to-face interview

(d) Assessment tool

**sFigure 3** Bubble plot of univariate meta-regression
